# Behavioral sciences in clinical practice

**DOI:** 10.1590/S1679-45082016ED3647

**Published:** 2016

**Authors:** Marcelo Katz, Hayden Barry Bosworth

**Affiliations:** 1 Hospital Israelita Albert Einstein, São Paulo, SP, Brazil.; 2 Departments of Medicine, Division of General Internal Medicine, Psychiatry and Behavioral Sciences, School of Nursing, Duke University Medical Center, Durham, NC, USA; Center for Health Services Research in Primary Care, Durham VAMC, Durham, NC, USA.

Suppose you have a patient sitting in front of you. She is a 58-year-old woman who works a lot and reports a high level of daily stress. She is hypertensive, smoker for more than 30 years, is overweight and has not been exercising regularly. She came to see you because of low back pain and poor sleep. As a healthcare professional, you want to help her. One major goal is to address the risk factors for cardiovascular disease, but you will also want to alleviate her current acute conditions. At this stage, you can simply follow the guidelines^1,2^ and give her advice on the benefits of exercise, weight loss, healthy eating and decreased sodium intake, inform her about atherosclerotic cardiovascular disease risks and prescribe medication on an evidence basis.

But will these recommendations really work? Do you take the patient through all of these topics, select a few, or let her direct the conversation? Will she adhere to your recommendations? And how can you know? What are the applicable incentives and penalties for complying or not with these guidelines? Are there other variables that should be included in this equation?

Behavioral sciences provide a starting point for healthcare professionals to address the questions raised above and improve health care delivery. In the past 70 years, scientists have developed and reframed a number of theories to explain the diverse patterns of human behavior, in different contexts and scenarios. Some of these theories can be combined and applied to daily clinical practice.

The health belief model is one of the best-known health related behavioral theories.^3,4^ The health belief model postulates that increased patient engagement in a given behavior (*e.g.*, exercising) results from the interaction between their personal beliefs about whether or not they are at risk for a given disease (*e.g*., increased risk of cardiovascular disease), their perception of exercise-related benefits (*e.g*., will exercise help me lose/maintain body weight?) and the barriers to taking action (*e.g*., spouse support, incorporation of exercise into daily routine) to decrease the chances of developing a serious health condition such as cardiovascular disease. Incorporating the health belief model into clinical practice requires effective communication with patients, so that usable information can be provided. However, identification of potential barriers and discussion of strategies to overcome them, along with the benefits of preventive actions, is also important. A challenge in the application of the health belief model is the mid- to long-term perception of health related benefits (*e.g*., the benefits of exercise may not be apparent for months), as opposed to the immediate perception of barriers (*e.g*., making time to exercise). The end result is the neglect of physical activity and procrastination when it comes to self-care.

The stages of changes, or transtheoretical model (TTM),^5^ is similar to the health belief model, but incorporates aspects of social cognitive theory (SCT; *e.g*., self-efficacy and the need to weigh pros and cons). In this model, patients are categorized into different stages of readiness to change behavior, as follows: (1) precontemplation: the patient does not consider taking action to change, there is no willingness to change; (2) contemplation: the patient shows interest in or begins to contemplate the possibility of changing; (3) preparation: the patient starts to work on a plan of action; (4) action: the patient changes and adopts a healthy behavior; (5) maintenance: healthy behavior is maintained for long period of time. This theory was initially conceptualized to address smoking and addiction, but can be applied in different settings. One aspect of TTM that may apply to clinicians is that, by assessing the patient’s stage of change, brief counseling can be provided to those in the precontemplative stage, and major efforts focused on those who are willing and have the necessary resources to engage in behavior changes straight away.

Mrs. K. complains of back stiffness and poor sleep. While these are the two most pressing issues from her perspective, you are concerned about her hypertension. You advise her on her stiffness and discuss how increasing her physical activity level may help with her pain, sleep, and blood pressure issues. Mrs. K. indicates that she would be willing to try some exercise – walking her dog in the morning. However, she is concerned about her occasional morning stiffness. You explore potential barriers and facilitators that may impact her exercising plans and ask how confident she is that she can start slowly and gradually increase her walking. You ask Mrs. K. to keep a record of her weekly exercise schedule and to try to walk every other day for a minimum of 15 minutes. She agrees to contact you in 4 weeks time to give you feedback on her exercise regimen and level of pain.

The third theory is SCT.^6^ According to SCT, personal and environmental factors continuously affect human behavior. Patients tend to learn from personal and other people’s experiences, and recalibrate their actions accordingly. Social support is a key component of SCT; therefore the group or team is an important player in the achievement of better individual outcomes. In this context, friends and family play a vital role in providing supporting to patients in health risk and disease scenarios.

Finally, self-efficacy, shared decisions and patient empowerment concepts must be emphasized. Self-efficacy can be defined as the confidence a patient has in his own ability to complete predetermined tasks and therefore reflects a dynamic belief, which can be improved when a task is accomplished and a positive feedback is given. Self-efficacy underpins all three theories discussed here and is one of the most reliable predictors of successful behavioral changes. In fact, when questioning patients about their level of confidence in their own ability to change a given behavior, anything below 7 in a 1-to-10 scale is likely to result in failure.^7-10^ Shared decisions and patient empowerment are also key to facilitate successful health care delivery, promote engagement and improve outcomes.

In conclusion, by applying behavioral sciences in clinical practice, the healthcare professional could inform the patient, thus sharpening the patient’s perception of his/her own cardiovascular risk; and refer the patient to a treatment center, thus empowering her. The healthcare professional is the coach and the patient is the main player, while family and friends are team members; together, the whole team can help her minimize her health risks. The healthcare professional could also assess her current ability to embrace behavioral changes, so that a targeted approach can be adopted. For example, the physician could determine whether patient is prepared to quit smoking and, if so, help her to plan accordingly. The physician could propose achievable weight loss goals based on incremental milestones, making sure positive feedback is given every time a milestone is achieved. Realistic goals within specific time frames must be set, and self-monitoring incorporated. Last but not least, stress issues must be addressed. Healthcare providers should try to understand her sources of stress and propose measures to help patients cope, such as mind-body therapies (*i.e*., yoga, meditation and exercise) and entertainment. As the biblical saying goes - *if you give a man a fish, he will eat today, but teach a man to fish and he will eat forever.*
