# Supplementary material for: Behavioral sciences in clinical practice
Source: Einstein (Sao Paulo). 2016 Jan-Mar;14(1):vii–xiv. doi: 10.1590/S1679-45082016ED3647 (PMC4872930; doi:10.1590/S1679-45082016ED3647)
Supplement: Translation

# Translation: Ciências do comportamento na prática clínica

**Authors:** Marcelo Katz, Hayden Barry Bosworth

**Affiliations:** 1 Hospital Israelita Albert Einstein, São Paulo, SP, Brasil.; 2 Departments of Medicine, Division of General Internal Medicine, Psychiatry and Behavioral Sciences, School of Nursing, Duke University Medical Center, Durham, NC, USA; Center for Health Services Research in Primary Care, Durham VAMC, Durham, NC, USA.

Suponha que você tenha uma paciente sentada a sua frente. Uma senhora de 58 anos, que trabalha muito e relata um alto nível de estresse diário. É hipertensa, tabagista há mais de 30 anos, está acima do peso e não faz atividades físicas regularmente. Queixa-se de dor lombar e dificuldade para dormir. Como profissional de saúde, você quer ajudá-la. Um dos principais objetivos é abordar os fatores de risco para doença cardiovascular, mas também considerar o quadro agudo. Nesse momento, você poderia simplesmente seguir as diretrizes^1,2^ e aconselhá-la sobre os benefícios da atividade física, da perda de peso, de uma alimentação saudável e da redução do consumo de sódio, além de dar informações sobre riscos da doença cardiovascular aterosclerótica e prescrever medicações com base em evidências científicas.

Mas será que essas recomendações surtiriam efeito? Você deve discutir todos esses assuntos com a paciente, selecionar alguns, ou deixar que ela conduza a conversa? Ela seguirá suas recomendações? Como você poderá saber? Quais os incentivos e desfechos cabíveis, em relação a cumprir ou não as diretrizes? Há outras variáveis que devam ser incluídas nessa equação?

As ciências do comportamento fornecem um ponto de partida para que profissionais de saúde possam abordar as questões acima e melhorar os cuidados prestados. Nos últimos 70 anos, os cientistas desenvolveram e reformularam diversas teorias para explicar os diferentes padrões de comportamento humano, em diferentes contextos e situações. Algumas dessas teorias podem ser combinadas e aplicadas à prática clínica diária.

O modelo de crenças em saúde é uma das mais bem conhecidas teorias comportamentais relacionadas à saúde^3,4^ e postula que o maior engajamento do paciente em um dado comportamento (atividade física, por exemplo) resulta da interação entre sua crença pessoal a respeito do próprio risco de desenvolver alguma doença (como maior risco de doença cardiovascular), sua percepção dos benefícios do exercício (o exercício pode me ajudar a perder/manter o peso?) e as barreiras que o impedem de agir (apoio do cônjuge, incorporação do exercício na rotina diária), no sentido de reduzir as chances de desenvolver doenças graves, como a doença cardiovascular. A incorporação do modelo de crenças em saúde na prática clínica requer uma comunicação efetiva com o paciente, de modo que informações úteis possam ser fornecidas. Entretanto, é também importante identificar as barreiras potenciais e discutir as estratégias para superá-las, além dos benefícios de ações preventivas. Um dos desafios envolvidos na aplicação do modelo de crenças em saúde é a percepção dos benefícios de saúde a médio/longo prazo (os benefícios do exercício podem demorar meses para aparecer), frente à percepção imediata das barreiras (conseguir tempo para se exercitar, por exemplo). Os resultados são a negligência da importância da atividade física e a procrastinação, quando se trata de autocuidado.

Os estágios das mudanças, ou modelo transteórico (MTT),^5^ é semelhante ao modelo da crenças em saúde, mas incorpora aspectos da teoria cognitiva social (TCS; por exemplo, autoeficácia e necessidade de pesar prós e contras para tomada de ações). De acordo com esse modelo, os pacientes são classificados em diferentes estágios de prontidão para mudança de comportamento, a saber: (1) pré-contemplação: o paciente não considera a mudança de comportamento e não há intenção de mudar; (2) contemplação: o paciente se interessa ou começa a contemplar a possibilidade de mudança; (3) preparação: o paciente começa a traçar um plano de ação; (4) ação: o paciente faz a mudança e adota um comportamento saudável; (5) manutenção: o comportamento saudável é mantido por longo período de tempo. Essa teoria foi inicialmente conceitualizada para abordar o tabagismo e o comportamento de vício, mas pode ser aplicada em diferentes situações. A avaliação do estágio de mudança de comportamento de um paciente permite ao médico fornecer um breve aconselhamento aos que se encontram no estágio pré-contemplativo, e concentrar esforços naqueles que se dispõem e têm os recursos necessários para mudar de comportamento de imediato.

Sra. K. queixa-se de rigidez na coluna e dificuldade para dormir. Embora sejam os dois problemas mais prementes na visão dela, você se preocupa com a hipertensão arterial sistêmica. Orienta sobre rigidez e argumenta que aumentar o nível de atividade física pode ajudá-la a controlar a dor e pressão arterial, além de melhorar o sono. Sra. K. se diz disposta a tentar se exercitar, levando o cão para passear pela manhã. Entretanto, preocupa-se com a eventual rigidez matinal. Você explora as potencias barreiras e os facilitadores que poderiam interferir nos planos de exercício da paciente, e pergunta se sente segurança para começar lentamente e aumentar as caminhadas de forma gradual. Você pede que faça um registro de seu programa semanal de exercícios e a aconselha a tentar andar por um mínimo de 15 minutos, em dias alternados. Ela concorda em contatá-lo dentro de 4 semanas para dar notícias sobre o programa de atividades físicas e o nível de dor.

A terceira teoria é a TCS,^6^segundo a qual fatores pessoais e ambientais afetam continuamente o comportamento humano. Os pacientes tendem a aprender com as próprias experiências e as alheias, equilibrando, assim, suas atitudes. O apoio social é um componente chave da TCS; consequentemente, o grupo ou equipe é um fator importante na obtenção de melhores resultados individuais. Nesse contexto, os amigos e familiares desempenham um papel fundamental, ao dar o apoio necessário em situações que envolvem risco à saúde e doenças.

Finalmente, os conceitos de autoeficácia, decisões compartilhadas e empoderamento do paciente devem ser destacados. A autoeficácia pode ser definida como a confiança que o paciente tem em sua própria capacidade de cumprir tarefas predeterminadas, que, consequentemente, reflete uma opinião dinâmica, que pode ser melhorada mediante *feedback* positivo, quando a tarefa é realizada. A autoeficácia é um elemento básico comum às três teorias discutidas aqui e constitui um dos preditores mais confiáveis de mudanças comportamentais bem-sucedidas. Na verdade, ao se questionar um paciente sobre seu nível de confiança na própria capacidade de mudar um determinado comportamento, qualquer nota abaixo de 7, em uma escala de 1 a 10, é um provável indicador de insucesso na mudança comportamental.^7-10^ As decisões compartilhadas e o empoderamento do paciente também são fundamentais para prestação de cuidados bem-sucedida, promover engajamento e melhorar resultados clínicos.

Concluímos que a aplicação das ciências do comportamento na prática clínica permitiria ao profissional de saúde informar a paciente, aumentando sua percepção dos próprios riscos cardiovasculares; e trazer a paciente para o centro do tratamento, empoderando-a. O profissional de saúde orienta, a paciente tem papel principal, enquanto a família e os amigos completam o time; juntos, todos podem ajudar a paciente a minimizar seus riscos de saúde. O profissional poderia também avaliar a atual capacidade da paciente para mudanças de comportamento, de modo que uma abordagem específica possa ser adotada. Por exemplo, o médico poderia determinar se ela está preparada para parar de fumar e, se for esse o caso, ajudá-la a traçar um plano. Poderia ainda propor os objetivos de perda de peso passíveis de serem alcançados e baseados em metas crescentes, certificando-se de que um *feedback* positivo seja dado toda vez que uma meta for atingida. Os objetivos devem ser realistas e encaixados em um período de tempo específico, além de incorporar o automonitoramento. Por último, mas não menos importante, a questão do estresse deve ser abordada. Tentar compreender as fontes de estresse da paciente e propor medidas que a ajudem a suportá-lo, como terapias mente-corpo (ioga, meditação e exercícios) e lazer. Como diz o provérbio bíblico − *dê um peixe a uma pessoa e ela comerá hoje; ensine-a a pescar, e ela comerá sempre*.
